# Case Report: Single-cell metabolic labeling probe for diagnosing renal tuberculosis

**DOI:** 10.3389/fmed.2026.1849446

**Published:** 2026-08-05

**Authors:** Yuanyuan Zou, Guiqin Dai, Huiling Liu, Jie Lin, Deliang Liu, Pengli Xu, Zhiqiang Lin, Xiafei Dai, Yang Zhou, Mingbin Zheng, Hongzhou Lu, Pengfei Zhao

**Affiliations:** 1 National Clinical Research Center for Infectious Disease, Shenzhen Third People’s Hospital, Southern University of Science and Technology, Shenzhen, China; 2 Molecular Biology Research Center and Center for Medical Genetics, School of Life Sciences, Central South University, Changsha, China; 3 The Affiliated Dongguan Songshan Lake Central Hospital, Guangdong Medical University, Dongguan, China; 4 Shenzhen Key Laboratory for Infectious Diseases, Shenzhen Third People’s Hospital, Shenzhen, China

**Keywords:** metabolic labeling, paucibacillary sample, renal tuberculosis, sensitive diagnosis, therapeutic monitoring

## Abstract

Extrapulmonary tuberculosis (EPTB) is characterized by low bacterial load and complex matrix in lesion samples. Therefore, current tuberculosis (TB) diagnostic methods often lose sensitivity to detect *Mycobacterium tuberculosis* (*Mtb*), leading to missed diagnosis and delayed treatment. Herein, we presented a single-cell metabolic labeling probe for tuberculosis (SCMLP-TB), which exhibited supporting investigative evidence for diagnosis of renal TB case, and enabled investigative monitoring of therapeutic efficacy of anti-TB regimens. A 51-year-old patient presented with fever, generalized weakness and muscle soreness on admission. Chest computed tomography (CT) revealed calcified mediastinal lymph nodes, and relevant renal function indicators were abnormal. Conventional TB diagnostic methods, including Xpert MTB/RIF, TB-DNA testing, and culture, yielded negative results using sputum, blood, and urine samples. Notably, SCMLP-TB assay detected *Mtb* in urine samples, suspecting renal TB. SCMLP-TB assay further detected gradually decreased bacterial viability along with anti-TB treatment (isoniazid, rifapentine, ethambutol, and pyrazinamide), revealing the efficacy of current regimen. The patient’s symptom resolution and anemia/renal function improvement provided auxiliary evidence of antimicrobial efficacy. Ultimately, SCMLP-TB showed great potential for non-invasive, sensitive detection of suspected renal TB, and enabled investigative monitoring of the therapeutic efficacy.

## 1 Introduction

Extrapulmonary tuberculosis (EPTB) accounts for 15–20% of tuberculosis (TB), low bacterial load and complex matrix in lesion samples making TB diagnosis difficult (1, 2). Renal TB is a common urinary tract TB, accounting for 5% of total EPTB cases. It has high misdiagnosis rate due to atypical symptoms and low bacterial burden, and delayed treatment often cause irreversible renal function impairment and long-term disability (3). For instance, salts and antimicrobial peptides in urine suppress bacterial viability and impede nucleic acid amplification, reducing detection sensitivity (4, 5). As a result, conventional diagnostic methods (smear microscopy, culture, and Xpert MTB/RIF) lose sensitivity to detect *Mtb* cells, leading to false-negative results and delayed treatment, which may further lead to infection exacerbation and pathophoresis (6). Furthermore, monitoring of anti-TB efficacy is still challenging. The golden standard for drug susceptibility testing (DST) requires 4 weeks for solid culture and additional 4 weeks for drug sensitivity test, which cannot feedback initial therapeutic efficacy timely (7). Lack of prompt therapeutic efficacy evaluation may cause hypermedication and severe adverse effects, including drug resistance, liver/kidney failure, and treatment failure (8). Therefore, a clinical accessible and matrix-resilient TB diagnostic technology with high sensitivity and therapeutic efficacy monitoring capabilities is urgently needed.

By reflecting bacterial viability characteristics, metabolic labeling strategies have emerged as a promising alternative for both TB diagnosis and real-time bacterial activity monitoring (9, 10). In this case report, a single-cell metabolic labeling probe for TB (SCMLP-TB) is introduced for investigational renal TB diagnosis. Composed of the mycomembrane (MM) metabolic substrate and aggregation-induced-emission fluorophore motif, the SCMLP-TB could integrate and aggregate on bacterial cytoderm, allowing for dynamic detection of live bacteria even in low-bacterial-load samples, facilitating rapid antimicrobial efficacy evaluation (11, 12). Unlike conventional molecular or culture-based methods, SCMLP-TB enables sensitive *Mtb* detection within approximately 2 h *via* metabolic labeling of viable bacteria. SCMLP-TB can realize dynamic detection of live bacteria with strong resistance to matrix in various samples, high sensitivity and rapid turnaround, thus providing a convenient and non-invasive solution for TB diagnosis (13–15). A 51-year-old female with unexplained fever for 2 months and concurrent renal impairment was presented on admission. The performance of SCMLP-TB for investigational renal TB diagnosis was compared with multiple conventional methods using sputum, blood and urine samples. *Via* monitoring the changes in *Mtb* fluorescence (FL) intensity, SCMLP-TB was utilized to indicate bacterial viability and therapeutic efficacy following anti-TB treatment. The patient’s symptom and anemia/renal function indicators were also investigated to evaluate treatment outcomes. This metabolic labeling technology showed great promise for sensitive detection and investigative monitoring of therapeutic efficacy for paucibacillary EPTB.

## 2 Case presentation

**Figure 1:**
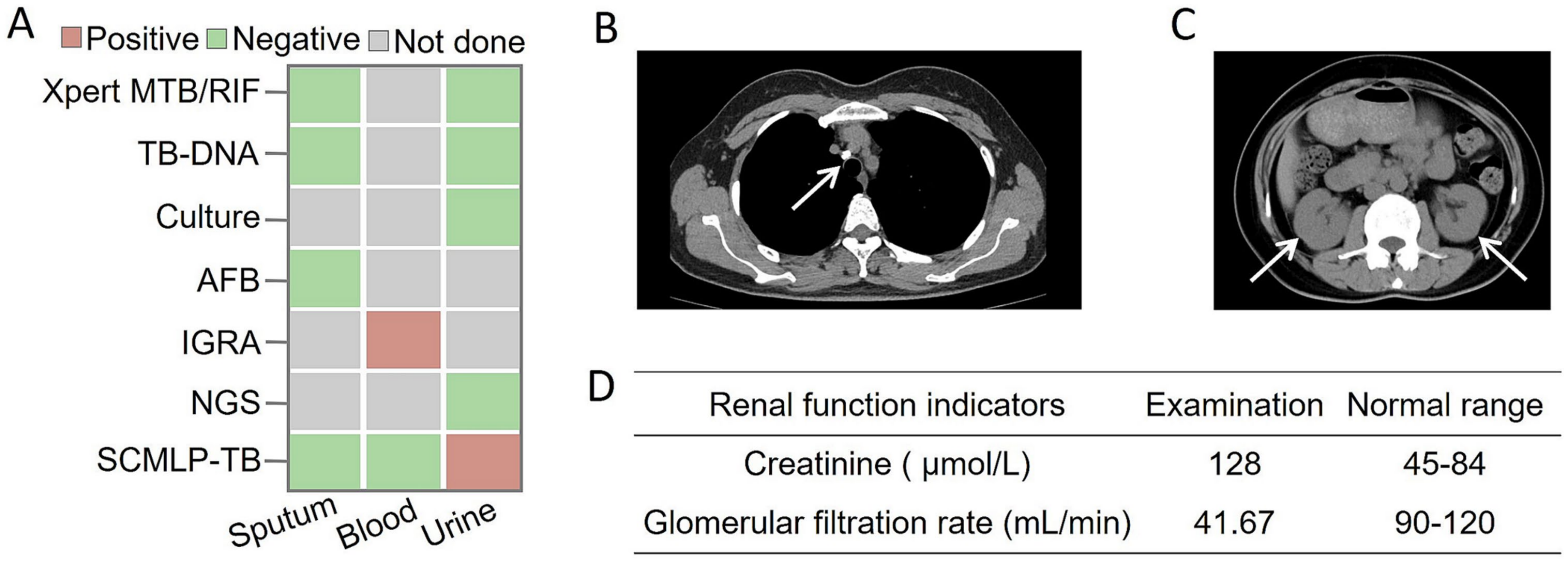
Clinical diagnostic results for the suspected renal TB case. **(A)** Multiple TB diagnostic methods failed to detect *Mtb* in sputum, blood, and urine samples from the suspected TB case, while SCMLP-TB detected *Mtb* pathogen in urine. All tests were completed on day 0. Sputum Xpert MTB/RIF, TB-DNA, AFB staining, and SCMLP-TB assays were all negative. Peripheral blood IGRA tested positive and SCMLP-TB negative. Urine Xpert MTB/RIF, TB-DNA, mycobacterial culture, and NGS were negative, while urine SCMLP-TB was positive. **(B)** Chest CT image illustrating calcified mediastinal and hilar nodes. The white arrow indicated the lymph node calcification. **(C)** Renal CT image showing no kidney abnormality. White arrows indicated kidneys. **(D)** Creatinine and glomerular filtration rate in patient’s peripheral blood after admission. TB-DNA, tuberculosis-deoxyribonucleic acid; AFB, acid fast bacilli; IGRA, interferon gamma release assay; NGS, next-generation sequencing.

A 51-year-old woman presented the Infectious Disease Out-patient at Shenzhen Third People’s Hospital. Fifty-four days pre-admission, the patient had experienced recurrent fever, generalized weakness, and muscle soreness. Subsequently, hepatorenal insufficiency and mild anemia, and a positive IGRA assay at an outside hospital, suggestive of *Mtb* infection. Before admission, the patient received conventional anti-infective therapy with ganciclovir, meropenem, and levofloxacin for 2 weeks, but showed no improvement in fever, generalized weakness or muscle soreness (Supplementary Figure S1). After admission, the patient reported no known contact with TB patients before and had no significant comorbidities. The patient was previously healthy, with otherwise unremarkable medical, family, and social histories. The patient denied any urinary symptoms (dysuria and frequency), and urinalysis was normal. Laboratory investigations showed blood C-reactive protein <0.6 mg/L, procalcitonin 0.06 ng/mL, erythrocyte sedimentation rate 16 mm/h, Alanine aminotransferase (ALT) 92 U/L and Aspartate Aminotransferase (AST) 72 U/L. Physical examination revealed stable vital signs (body temperature 36.6 °C, pulse 60 beats per minute, blood pressure 121/69 mmHg) with no other remarkable findings. Given the symptoms suggestive of TB, systematic TB diagnostics were performed using sputum, blood, and urine samples, including Xpert MTB/RIF, TB-DNA, culture, acid-fast bacillus (AFB) testing, and interferon-gamma release assay (IGRA). The IGRA result in blood was positive, while conventional diagnostic methods for TB were unable to confirm TB (Figure 1A). Because of the paucibacillary nature of renal TB, conventional diagnostic methods failed to detect *Mtb* (6). Noteworthy, chest computed tomography (CT) images showed calcified mediastinal lymph nodes, a classic radiographic sign of recovered prior TB, indicating a history of previous TB infection (Figure 1B). The patient also presented renal dysfunction (serum creatinine 128 μmol/L, glomerular filtration rate 41.67 mL/min). Renal CT images showed no evidence of nephrolithiasis, masses or hydronephrosis (Figures 1C,D).

**Figure 2:**
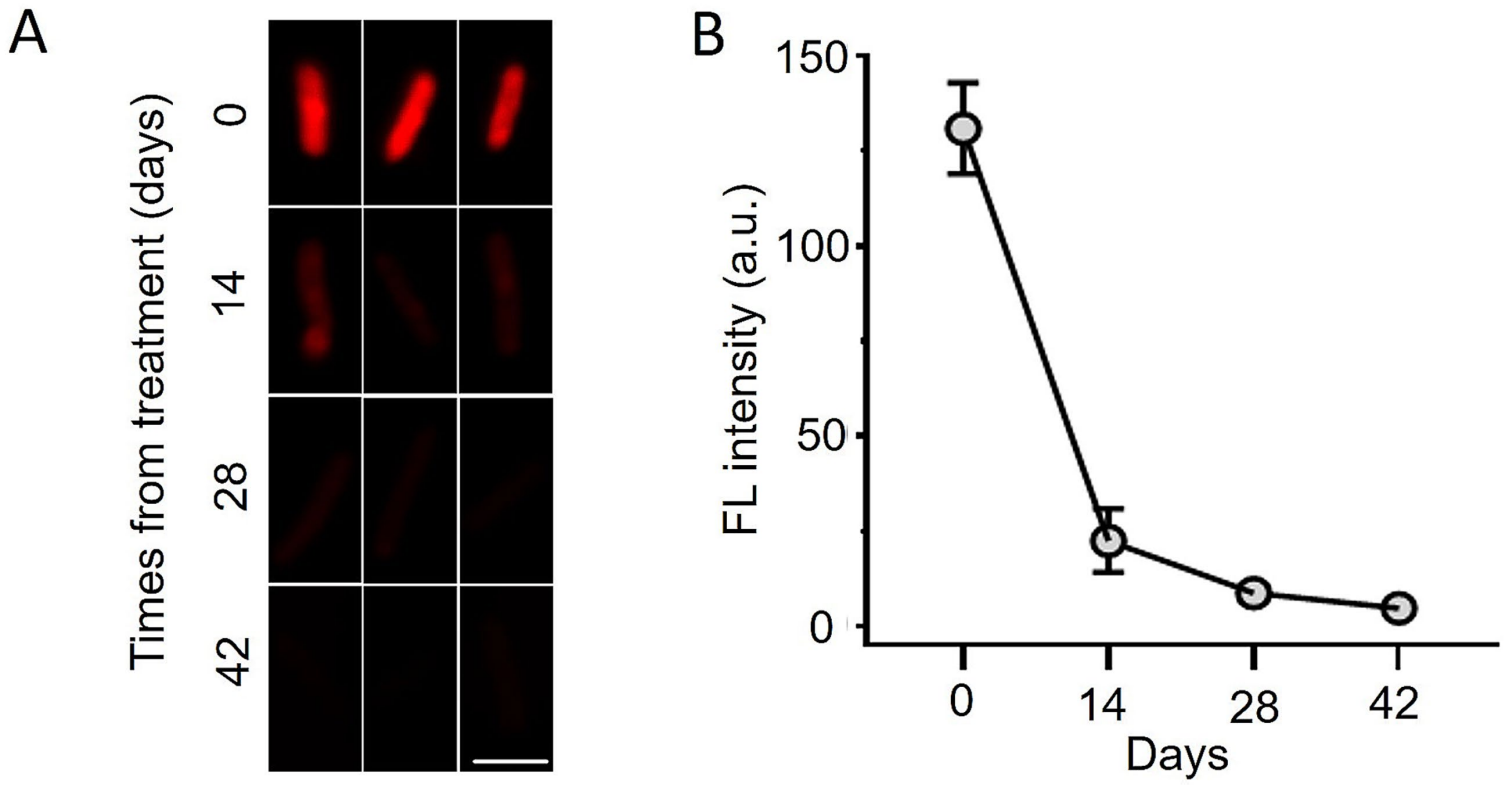
*Mtb* detection results in urine during treatment using SCMLP-TB. **(A)** Single *Mtb* images in urine using the SCMLP-TB assay on day 0, day 14, day 28, and day 42 post anti-TB treatment. *Mtb* cells were presented in red. Scale bar = 2 μm. **(B)** Changes in *Mtb* fluorescence (FL) intensity over the course of anti-TB treatment. Treatment regimen: isoniazid, rifapentine, ethambutol, and pyrazinamide. Anti-TB therapy was initiated on day 0. The labeled *Mtb* was visualized under a Zeiss LSM 980 confocal microscope (63× oil immersion objective, 480 nm excitation), and fluorescence intensity was quantified by ZEN 3.6 software (*n* = 3 cells per group). Three *Mtb* cells were selected from the patient’s urine sample at each time point for technical observation. Data were shown as mean ± SD, which were descriptive summaries of technical cell observations.

As reported in our previous studies, the SCMLP-TB exhibited labeling of viable *Mtb* and a low detection limit of 10 CFU/mL, and demonstrated a diagnostic sensitivity and specificity of 97.5 and 96.7% for clinical TB (13, 15). Notably, the sensitivity of SCMLP-TB was superior to culture (62.5%) and Xpert MTB/RIF (72.5%), showing great potential for paucibacillary sample detection (13). Therefore, we collected the patient’s sputum, blood, and urine samples and applied the SCMLP-TB probe for diagnosis. Briefly, midstream morning urine (20 mL) was filtered through a 40 μm cell strainer to remove large debris, followed by centrifugation at 4,000 rpm for 10 min. Sputum samples were digested using Sodium Hydroxide-N-Acetyl-L-Cysteine followed by filtration and centrifugation, while blood samples were subjected to centrifugation after red blood cell lysis. Afterwards, the samples were resuspended, and 100 μL of the concentrated samples were incubated with the SCMLP-TB (40 μM) at 37 °C for 2 h. After washing with PBS, samples were centrifuged and subjected to fluorescence imaging using a ZEISS LSM 980 confocal microscope (63× oil immersion objective, 480 nm excitation) (Carl Zeiss, GER). The fluorescence intensity of labeled bacteria was quantified using ZEN 3.6 software (*n* = 3 cells per group). The positivity threshold and the limit of detection (LOD) (10 CFU/mL) were based on our previously validated study (13, 15). *Mtb* H37Ra was set as positive control, and heat-inactivated *Mtb* H37Ra was served as negative controls. Operator blinding corresponds to single-blind design. The SCMLP-TB could incorporate into the glycolipid layer *via* Ag85 acyltransferase catalysis and aggregate on *Mtb* cytoderm, realizing single-bacterium metabolic labeling of *Mtb* cells. Following SCMLP-TB assay, brightly fluorescently labeled *Mtb* cells were visualized by fluorescence microscopy in the urine sample (Figures 1A, 2A). Due to the absence of independent microbiological confirmation, suspected renal TB was formulated in compliance with the following diagnostic criteria: (1) Typical clinical manifestations including low-grade fever and urinary system abnormalities; (2) Serological and immunological evidence of TB infection confirmed by positive IGRA; (3) Positive SCMLP-TB detection results indicating *Mtb* in renal tissues. Meanwhile, common alternative conditions such as other occult uropathogenic infections, urolithiasis, chronic nephritis and renal space-occupying diseases were comprehensively ruled out *via* laboratory tests and imaging examinations.

## 3 Treatment follow-up and outcome

The maximum doses of medication used by this patient during treatment were isoniazid (0.6 g/day), rifapentine (0.6 g/week), ethambutol (0.75 g/day), and pyrazinamide (1.5 g/day). The planned regimen consisted of a 2-month intensive phase followed by a 4-month continuation phase. The therapeutic dosages are utilized according to the World Health Organization ‘Comprehensive Guidelines’ and the 2023 Chinese Expert Consensus (16–18). Pyridoxine is the prescribed drug for patients with high risk of isoniazid-induced neuropathy, including those with diabetes or malnutrition. But this patient had no relevant risk factors, so pyridoxine was not applied (19). Liver function, renal function, complete blood count, and visual symptoms were monitored regularly during treatment, and serum uric acid was assessed every 2 months. During treatment, the patient demonstrated good adherence and tolerability, and no treatment-related adverse events were observed. Drug dosing was guided by body weight, serial clinical assessments and multidisciplinary consultations.

**Figure 3:**
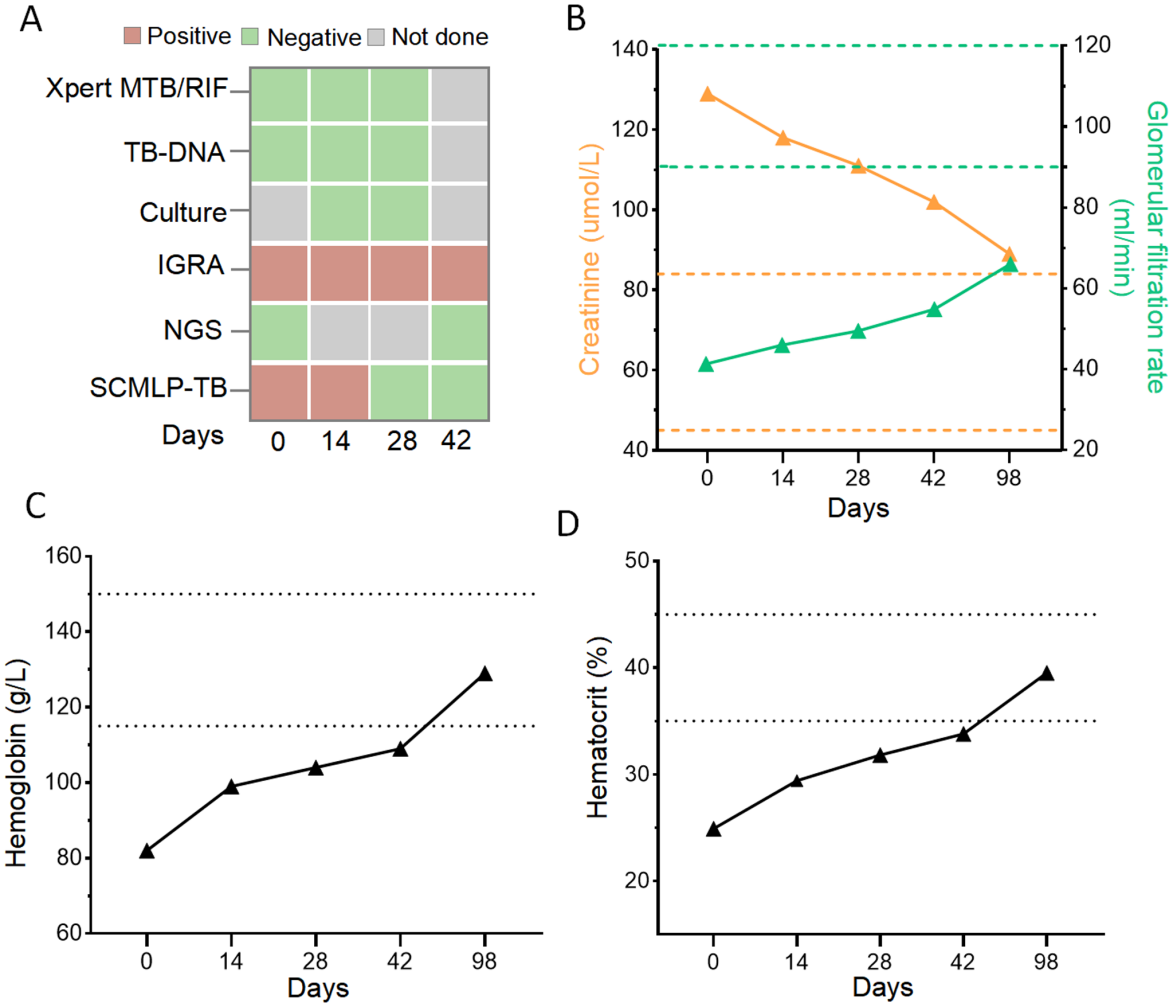
Diagnosing *Mtb* in urine and treatment follow-up. **(A)** Diagnostic results of conventional diagnostics and SCMLP-TB throughout anti-TB treatment using urine samples. **(B)** Creatinine and glomerular filtration rate of the patient before and after treatment. The normal creatinine range was 45–84 μmol/L, and the normal GFR range was 90–120 mL/min. **(C,D)** Hemoglobin and hematocrit levels in patient’s peripheral blood before and after treatment. The normal hemoglobin range was 115–150 g/L, and the normal hematocrit range was 35–45%. The reference ranges for creatinine, glomerular filtration rate, hemoglobin, and hematocrit were within the normal dashed line areas.

During treatment, we used SCMLP-TB assay to detect *Mtb* in urine samples every 14 days. Fluorescence signals decreased gradually on day 14, 28, and 42, and no signal could be detected on day 42 (Supplementary Figure S1; Figure 2B). Other conventional diagnostic methods for TB, including Xpert MTB/RIF, AFB, TB-DNA, culture, and NGS, still failed to monitor *Mtb* existence in urine samples during treatment (Figure 3A). Meanwhile, we monitored clinical indicators of anemia (hemoglobin and hematocrit) and renal function (creatinine and glomerular filtration rate) during anti-TB treatment. Follow-up results after 98 days of treatment showed gradual improvement in clinical indicators of anemia and renal function, indicating that the current anti-tuberculosis treatment regimen was effective (Figure 3B). Moreover, hemoglobin and hematocrit levels returned to normal levels, and the patient’s clinical symptoms improved, with resolution of fever and limb weakness (Figures 3C,D). Ultimately, SCMLP-TB assay provided supporting investigative evidence for diagnosis of TB from low-bacterial-load extrapulmonary samples, and enabled investigative monitoring of therapeutic efficacy, thereby providing key information to support clinical decision-making. The patient expressed that she understood the diagnosis of suspected renal TB and accepted the proposed treatment. During treatment, the patient demonstrated good adherence and tolerability, and no treatment-related adverse events were observed. Follow-up evaluation in last September confirmed recovery of renal function parameters and resolution of all clinical symptoms.

## 4 Discussion

Current diagnostic techniques show markedly reduced sensitivity to diagnose extrapulmonary tuberculosis (EPTB) due to extremely low bacterial load and complex matrix, increasing the risk of delayed treatment (2, 20). For example, urine contains various proteins and cellular debris that can interfere with PCR amplification and bacterial detection (21). The near-infrared SCMLP-TB can act as a metabolic substrate that incorporates and aggregates on the cell wall, enabling single-bacterium labeling without matrix interference (22, 23). Moreover, SCMLP-TB exhibited poor labeling capability to multiple non-tuberculous mycobacteria, such as *Mycobacterium kansasii* and *Mycobacterium avium*, and other common pathogenic bacteria such as *Staphylococcus aureus* and *Escherichia coli* (15). Ultimately, the SCMLP-TB enabled detection of *Mycobacterium tuberculosis* (*Mtb*) in urine to provide supporting investigative evidence for diagnosis of renal TB. Since SCMLP-TB was an assay under evaluation, it could not serve as an independent reference standard. But this technique provided a sensitive and matrix-resilient single-cell framework for EPTB such as renal TB.

Delayed feedback on anti-TB drug efficacy usually results in excessive medication and drug resistance, leading to increased adverse effects and treatment failure (24). Culture-based phenotypic drug susceptibility testing takes up to 56 days, whereas rapid molecular diagnostics such as Xpert MTB/RIF are limited to detecting known resistance genes and frequently show discordance with actual drug sensitivity (25, 26). Based on metabolic labeling technology, the SCMLP-TB assay enabled investigative monitoring of therapeutic efficacy in urine *via* fluorescence intensity, rapidly reflecting bactericidal effects of clinical anti-TB regimen. Therefore, SCMLP-TB provided timely drug efficacy feedback and accelerated the optimization of anti-TB regimens.

In summary, SCMLP-TB provided a sensitive and non-invasive diagnostic approach for suspected renal TB diagnosis *via* metabolic labeling. It also realized investigative monitoring of the therapeutic efficacy of anti-TB drugs, evidenced by the patient’s symptom resolution and anemia/renal function improvement. As this study reports a single case of suspected renal TB supported by SCMLP-TB and clinical response, which lacks independent microbiological confirmation of renal TB, the diagnostic performance and generalizability of SCMLP-TB remain to be established. In future, large-scale prospective cohort studies including diverse patients with suspected renal tuberculosis is needed to compare its sensitivity, specificity, reproducibility, and clinical utility with conventional methods, such as Xpert MTB/RIF, smear, and culture. In future, a large-scale, multicenter cohort study is needed to investigate SCMLP-TB’s generalizability for TB diagnosis, especially for low-bacterial-load patients (such as pediatric TB and HIV co-infection patients). Integrating artificial intelligence-based diagnosis techniques and portable detection devices, SCMLP-TB may enable TB screening, diagnosis and antimicrobial efficacy monitoring after validation.

## Data Availability

The original contributions presented in the study are included in the article/Supplementary material, further inquiries can be directed to the corresponding author.

## References

[1] Tulu B Brehm TT van Crevel R Dallenga T DiNardo AR Dheda K . Host- and pathogen-related determinants of pulmonary versus extrapulmonary tuberculosis. Eur Respir Rev. (2026) 35:250174. doi: 10.1183/16000617.0174-2025, 41605541 PMC12873656

[2] Luukinen B Ahava M Aittoniemi J Miikkulainen-Lahti T Pätäri-Sampo A. Diagnostic performance of Xpert MTB/RIF ultra assay with pulmonary and extrapulmonary specimens: a retrospective evaluation in a low-incidence setting in Finland. Clin Microbiol Infect. (2026) 32:87–94. doi: 10.1016/j.cmi.2025.09.002, 40945716

[3] Chaker K Gharbia N Zehani A Mosbahi B Zribi S Nouira Y. Renal tuberculosis mimicking cystic renal cell carcinoma: a case report. Urol Case Rep. (2025) 60:103001. doi: 10.1016/j.eucr.2025.103001, 40129534 PMC11930736

[4] Schott CG Kuester M Gruhlke MCH Slusarenko AJ Rühl M. Evaluation of molecular inhibitors of loop-mediated isothermal amplification (LAMP). Sci Rep. (2024) 14:5916. doi: 10.1038/s41598-024-55241-z 38467647 PMC10928092

[5] Kohli M Inbaraj LR Salomon A Scandrett K Korobitsyn A Ismail N . Low-complexity automated nucleic acid amplification tests for extrapulmonary tuberculosis and rifampicin resistance in adults and adolescents. Cochrane Database Syst Rev. (2025) 8:CD012768. doi: 10.1002/14651858.CD012768.pub4, 40757508 PMC12320217

[6] Wang MQ Zheng YF Hu YQ Huang JX Yuan ZX Wu ZY . Diagnostic accuracy of Xpert MTB/RIF ultra for detecting pulmonary tuberculosis and rifampicin resistance: a systematic review and meta-analysis. Eur J Clin Microbiol Infect Dis. (2025) 44:681–702. doi: 10.1007/s10096-024-05032-1, 39754613

[7] Domínguez J Boeree MJ Cambau E Chesov D Conradie F Cox V . Clinical implications of molecular drug resistance testing for *Mycobacterium tuberculosis*: a 2023 TBnet/RESIST-TB consensus statement. Lancet Infect Dis. (2023) 23:e122–37. doi: 10.1016/s1473-3099(22)00875-1, 36868253 PMC11460057

[8] The Lancet Infectious Diseases. New advances for drug-resistant tuberculosis. Lancet Infect Dis. (2025) 25:947. doi: 10.1016/s1473-3099(25)00485-2 40846442

[9] Smelyansky SR Ma CW Marando VM Babunovic GH Lee SY Bryson BD . Exploiting thioether reactivity to label mycobacterial glycans. Proc Natl Acad Sci USA. (2025) 122:e2422185122. doi: 10.1073/pnas.2422185122, 40324093 PMC12088412

[10] Liu S Li Y Yang Y Li X Wang L Xiao X . Lateral flow analysis test strips based on aggregation-induced emission technique: principle, design, and application. Biosens Bioelectron. (2025) 272:117058. doi: 10.1016/j.bios.2024.117058, 39746282

[11] Dai G Luo Y Liao M Zhang P Pan H Yin T . A cytoderm metabolic labeling AIEgen for rapid detection and intracellular ablation of *Mycobacterium tuberculosis*. Cell Rep Phys Sci. (2023) 4:101238. doi: 10.1016/j.xcrp.2022.101238

[12] Marando VM Kim DE Calabretta PJ Kraft MB Bryson BD Kiessling LL. Biosynthetic glycan labeling. J Am Chem Soc. (2021) 143:16337–42. doi: 10.1021/jacs.1c07430, 34606245 PMC8943913

[13] Liang G Dai G Hu X Liu D Lin Z Yang M . Cytoderm metabolic-labeling SCMLP-TB for pulmonary tuberculosis diagnosis: a preliminary diagnostic accuracy study. Drug Discov Ther. (2026) 20:147–54. doi: 10.5582/ddt.2025.01123, 41951372

[14] Yang M Dai G Li D Zhao P Zhan S Qin H . A cytoderm metabolic labeling TPAPy-Tre for real-time detection of vitality of *Mycobacterium tuberculosis* in sputum. Microbiol Spectrum. (2025) 13:e0245724. doi: 10.1128/spectrum.02457-24, 40401971 PMC12211060

[15] Dai G Zhao P Zhou Y Zhan S Liu D Liao M . Culture-free metabolic labeling analysis for point-of-care antimicrobial efficacy evaluation of tuberculosis. ACS Sens. (2026) 11:3616–27. doi: 10.1021/acssensors.5c02865, 42007846

[16] World Health Organization. Concept note dose optimization of rifampicin, isoniazid, pyrazinamide and ethambutol in the treatment of drug-susceptible tuberculosis (2024). Available online at: https://cdn.who.int/media/docs/default-source/hq-tuberculosis/public-calls/conceptnote_fld_tag_2024_rfp_final.pdf?sfvrsn=d206eea4_3 (Accessed July 03, 2026).

[17] Chinese Society for Tuberculosis, Chinese Medical Association. Zhonghua Jie He He Hu Xi Za Zhi. (2023) 46:1085–102. doi: 10.3760/cma.j.cn112147-20230809-00062 37914420

[18] Senior Department of Tuberculosis, The 8th Medical Center of Chinese PLA General Hospital, Editorial Board of Chinese Journal of Antituberculosis, Speciality Committee of Tuberculosis Control Branch of China International Exchange and Promotive Associatio. Expert consensus on diagnosis and treatment of urological tuberculosis. Chin J Antituberc. (2025) 47:546–58. doi: 10.19982/j.issn.1000-6621.20250058

[19] Nahid P Dorman SE Alipanah N Barry PM Brozek JL Cattamanchi A . Executive summary: official American thoracic society/centers for disease control and prevention/infectious diseases society of America clinical practice guidelines: treatment of drug-susceptible tuberculosis. Clin Infect Dis. (2016) 63:853–67. doi: 10.1093/cid/ciw566, 27621353 PMC6366011

[20] Theobald SJ Dahm K Lange D Spintge JB Winter S Klingmüller A . Deep immune profiling delineates hallmarks of disease heterogeneity in extrapulmonary tuberculosis. Nat Commun. (2025) 16:9662. doi: 10.1038/s41467-025-65561-x, 41213960 PMC12603278

[21] Malhotra AG Lokhande L Pakhare A Soni P Vishwakarma SP Maurya AK . Diagnostic yield of Xpert MTB/RIF, Xpert MTB/RIF ultra, and Truenat MTB assays on non-pulmonary samples from suspected cases of extra-pulmonary tuberculosis (EPTB). Eur J Clin Microbiol Infect Dis. (2025) 44:2093–103. doi: 10.1007/s10096-025-05177-7, 40448889

[22] Dai G Zhao P Song L He Z Liu D Duan X . Devising novel near-infrared aggregation-induced-emission luminogen labeling for point-of-care diagnosis of *Mycobacterium tuberculosis*. Biosci Trends. (2023) 17:234–8. doi: 10.5582/bst.2023.01087, 37245987

[23] Huang X Chu C Shi C Zhang J Yan B Shan F . Seeing is believing: efficiency evaluation of multifunctional ionic-dependent AIEgens for tuberculosis. Biomaterials. (2023) 302:122301. doi: 10.1016/j.biomaterials.2023.122301, 37690379

[24] Mdlenyani L Mohamed Z Stadler JAM Mtwa N Meintjes G Warren R . Treatment outcomes of bedaquiline-resistant tuberculosis: a retrospective and matched cohort study. Lancet Infect Dis. (2025) 25:1149–58. doi: 10.1016/s1473-3099(25)00218-x, 40543523 PMC12662777

[25] Zhang F Jiang J McBride M Yang Y Mo M Iriya R . Direct antimicrobial susceptibility testing on clinical urine samples by optical tracking of single cell division events. Small. (2020) 16:e2004148. doi: 10.1002/smll.202004148, 33252191 PMC7770081

[26] Hong R Xu P Lai W Wu C Zhang X Chen S . Discrepancy between WGS-based genotypic and phenotypic drug susceptibility testing of first and second-line drugs for *Mycobacterium tuberculosis*: limited impact of unknown resistance mechanisms. Int J Antimicrob Agents. (2026) 67:107666. doi: 10.1016/j.ijantimicag.2025.107666, 41224151

