# Supplementary material for: Case Report: Single-cell metabolic labeling probe for diagnosing renal tuberculosis
Source: Front Med (Lausanne). 2026 Aug 5;13:1849446. doi: 10.3389/fmed.2026.1849446 (PMC13486178; doi:10.3389/fmed.2026.1849446)
Supplement: Supplementary file 1 [file Image_1.pdf]

Supplementary Material

Case Report: Single-Cell Metabolic Labeling Probe for Diagnosing Renal Tuberculosis

Yuanyuan Zou, Guiqin Dai, Huiling Liu, Jie Lin, Deliang Liu, Pengli Xu, Zhiqiang Lin, Xiafei Dai, Yang Zhou*, Mingbin Zheng*, Hongzhou Lu*, Pengfei Zhao*

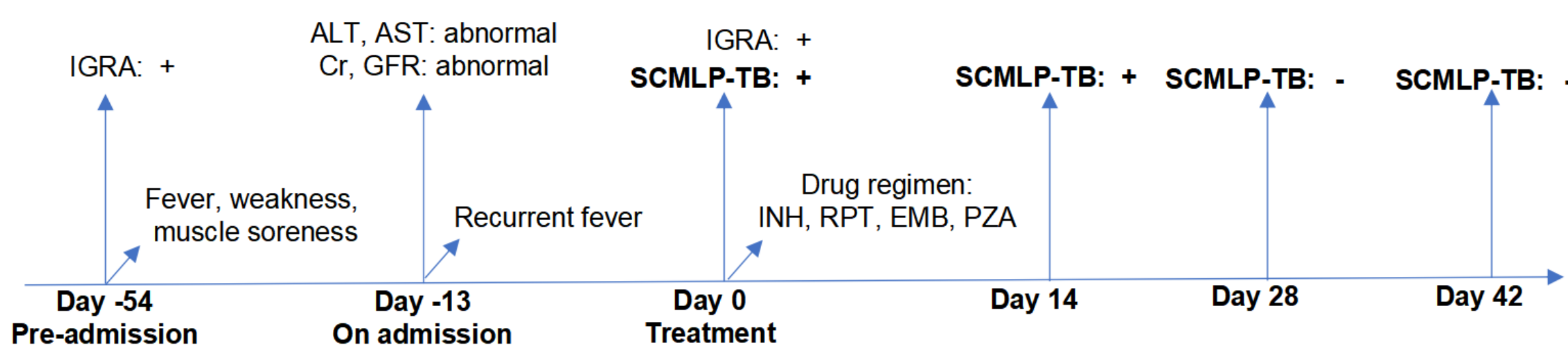

Figure S1. Timeline of the renal TB case. The timeline covered the patient's symptom onset, diagnostic testing, treatment initiation and follow-up. IGRA: Interferon-Gamma Release Assay. ALT: Alanine Aminotransferase. AST: Aspartate Aminotransferase. Cr: Creatinine. GFR: glomerular filtration rate. SCMLP-TB: single cell metabolic labeling probe for tuberculosis. INH: isoniazid. RPT: rifapentine. EMB: ethambutol. PZA: pyrazinamide.
